# Artificial Intelligence in Endoscopic and Ultrasound Imaging for Inflammatory Bowel Disease

**DOI:** 10.3390/jcm14124291

**Published:** 2025-06-16

**Authors:** Rareș Crăciun, Andreea Livia Bumbu, Vlad Andrei Ichim, Alina Ioana Tanțău, Cristian Tefas

**Affiliations:** 1 Department of Internal Medicine, “Iuliu Hațieganu” University of Medicine and Pharmacy, 8 Victor Babes Street, 400012 Cluj-Napoca, Romania; craciun.rares.calin@elearn.umfcluj.ro (R.C.); andreealiviabumbu@gmail.com (A.L.B.); ichimvlad@gmail.com (V.A.I.); alitantau@gmail.com (A.I.T.); 2 Department of Gastroenterology, “Prof. Dr. Octavian Fodor” Institute of Gastroenterology and Hepatology, 19-21 Croitorilor Street, 400162 Cluj-Napoca, Romania; 3 Department of Gastroenterology, 1st Medical Clinic, 3-5 Clinicilor Street, 400006 Cluj-Napoca, Romania; 4 Department of Gastroenterology, 4th Medical Clinic, 18 Republicii Street, 400015 Cluj-Napoca, Romania

**Keywords:** inflammatory bowel disease, artificial intelligence, endoscopy, video capsule endoscopy, intestinal ultrasound, Crohn’s disease, ulcerative colitis

## Abstract

Artificial intelligence (AI) is rapidly transforming imaging modalities in inflammatory bowel disease (IBD), particularly in endoscopy and ultrasound. Despite their critical roles, both modalities are challenged by interobserver variability, subjectivity, and accessibility issues. AI offers significant potential to address these limitations by enhancing lesion detection, standardizing disease activity scoring, and supporting clinical decision-making. In endoscopy, deep convolutional neural networks have achieved high accuracy in detecting mucosal abnormalities and grading disease severity, reducing observer dependency and improving diagnostic consistency. AI-assisted colonoscopy systems have also demonstrated improvements in procedural quality metrics, including adenoma detection rates and withdrawal times. Similarly, AI applications in intestinal ultrasound show promise in automating measurements of bowel wall thickness, assessing vascularity, and distinguishing between inflammatory and fibrotic strictures, which are critical for tailored therapy decisions. Video capsule endoscopy has likewise benefited from AI, reducing interpretation times and enhancing the detection of subtle lesions. Despite these advancements, implementation challenges, including dataset quality, standardization, AI interpretability, clinician acceptance, and regulatory and ethical considerations, must be carefully addressed. The current review focuses on the most recent developments in the integration of AI into experimental designs, medical devices, and clinical workflows for optimizing diagnostic accuracy, treatment strategies, and patient outcomes in IBD management.

## 1. Introduction

Inflammatory bowel diseases (IBD), encompassing Crohn’s disease (CD) and ulcerative colitis (UC) differ in genetic, epidemiological, clinical, endoscopic, and histopathological aspects, as well as in disease course [1]. They require precise and continuous evaluations for accurate diagnosis, monitoring of disease activity, and assessment of treatment response.

Endoscopy and ultrasound (US) are two essential imaging modalities widely employed in IBD management. Endoscopy provides direct visualization of the mucosa, allowing for histological sampling and detailed lesion characterization, while US, particularly bowel ultrasound, serves as a non-invasive tool capable of assessing transmural inflammation and complications such as strictures and abscesses. Together, these modalities play a pivotal role in clinical decision-making, guiding therapeutic adjustments, and predicting disease progression. Disease severity and activity are commonly assessed using standardized scoring systems such as the Mayo Endoscopic Score (MES) for UC and the Simple Endoscopic Score for CD (SES-CD) [2]. These indices rely on endoscopic findings such as ulceration, vascular pattern loss, and mucosal damage. In ultrasound evaluation, parameters such as bowel wall thickness, vascularity (Doppler signal), and the presence of complications are used to generate structured scoring systems, facilitating objective assessment [3].

Despite their utility, both endoscopy and US are subject to inherent limitations that can affect diagnostic accuracy and clinical outcomes. One major challenge is interobserver variability, as the interpretation of findings heavily relies on the expertise and experience of the examiner [4,5]. Discrepancies in scoring endoscopic disease severity or assessing bowel wall thickness on ultrasound can lead to inconsistencies in treatment decisions. Moreover, image interpretation is often time-consuming, requiring meticulous review and correlation with clinical and laboratory data. The subjectivity in lesion assessment further complicates standardization, impacting the reproducibility of disease activity scores across different healthcare settings. Additionally, accessibility to expert-level imaging interpretation may be limited in certain regions, leading to disparities in patient care. These challenges highlight the need for improved imaging solutions to optimize IBD evaluation and management.

Artificial intelligence (AI) has emerged as a transformative tool, offering solutions to overcome the limitations associated with endoscopy and US. AI-driven algorithms have demonstrated significant potential in lesion detection, disease activity scoring, and predictive analytics [6,7,8]. In endoscopy, deep learning models can automate the identification of ulcers, erosions, and inflammatory patterns, reducing observer dependency and improving diagnostic consistency [9,10]. Similarly, AI-enhanced US applications can refine the assessment of bowel wall thickening, vascularity, and transmural lesions, contributing to a more objective and reproducible evaluation [11,12]. Machine learning models can also aid in distinguishing between active inflammation and fibrosis, a critical distinction in guiding therapy decisions [13,14].

Furthermore, AI-powered predictive analytics could integrate imaging data with clinical parameters to forecast disease flares, therapy response, and long-term outcomes, enabling proactive patient management. By incorporating large-scale datasets and real-time analysis, AI has the potential to provide rapid and accurate assessments, reducing diagnostic delays and improving treatment strategies. AI can also support automated segmentation of imaging data, expediting workflows and minimizing the burden on clinicians. As AI technologies continue to evolve, their integration into IBD workflows has the potential to enhance diagnostic precision, reduce clinician workload, and improve patient care.

Several recent reviews have explored the potential of AI in IBD imaging. However, many are narrowly focused (e.g., limited to colonoscopy or ultrasound) or lack a detailed clinical contextualization of how AI tools may be integrated into patient management strategies. This review aims to fill the following gaps:

1. Multimodal Integration: Unlike prior reviews that separately discuss endoscopy or US, this manuscript presents a cohesive overview of AI applications across three major imaging domains: conventional endoscopy, video capsule endoscopy, and intestinal ultrasound.
2. Workflow-Oriented Perspective: We evaluate how AI augments not only diagnostic accuracy but also procedural quality metrics (e.g., effective withdrawal time, bowel prep scores, real-time quality control), which are underrepresented in existing reviews.
3. Real-World Implementation Challenges: By highlighting barriers such as data heterogeneity, AI model explainability, and clinician acceptance, this review moves beyond algorithm performance to practical considerations for clinical adoption—particularly relevant as regulatory frameworks for AI tools in gastroenterology evolve.
4. Standardization and Validation Emphasis: We systematically appraise which models have been externally validated or tested in multicenter trials, drawing attention to studies with robust methodology and clinical relevance.
5. Emerging Concepts: This review introduces novel AI-guided concepts such as endo-omics, AI-driven fibrosis assessment, and CEUS parameter modeling, highlighting their potential to guide therapeutic decisions and longitudinal monitoring.

The next sections of this review will address these gaps and present the latest developments in AI applications for endoscopic and US-based IBD assessment, highlighting their impact on clinical practice and future directions in the field.

## 2. Methods

This review was conducted with the aim of synthesizing the most recent and relevant evidence regarding the integration of AI into endoscopic and ultrasound imaging in IBD. A literature search was performed using PubMed, Scopus, and Web of Science databases from January 2018 through March 2025. Search terms included combinations of “artificial intelligence,” “machine learning,” “deep learning,” “endoscopy,” “ultrasound,” “capsule endoscopy,” “Crohn’s disease,” and “ulcerative colitis.” Only English-language articles were included.

We focused on peer-reviewed original research studies, systematic reviews, meta-analyses, and technical validation reports that specifically addressed AI applications in image-based diagnostics or monitoring of IBD. Studies were eligible if they involved clinical cohorts, phantom models, or AI algorithm validation using annotated datasets related to IBD imaging. Case reports and studies with non-human data were excluded.

The quality of included studies was evaluated based on criteria adapted from the QUADAS-2 tool for diagnostic accuracy studies and the CLAIM checklist for AI model reporting. Specifically, we assessed the clarity of inclusion criteria and patient selection, dataset size and diversity, AI model transparency and performance metrics (AUROC, sensitivity, specificity), and prospective validation or external dataset testing.

Studies with high risk of bias or poor reporting were discussed with critical commentary rather than excluded, given the evolving nature of AI in clinical research. Where available, we prioritized studies that demonstrated real-world applicability, external validation, or integration with clinical workflows.

The authors acknowledge the use of ChatGPT 4.5 (OpenAI, San Francisco, CA, USA) and Grammarly Premium Editor (Grammarly Inc., San Francisco, CA, USA) for phrasing, language polishing, and structural feedback. All of the primary data included in the manuscript have been written and verified by the authors. The responsibility for the content, including the parts produced by the AI tools, lies entirely with the authors, and the authors are liable for any breach of publication ethics. 

## 3. Artificial Intelligence in Endoscopy

### 3.1. Endoscopic Lesion Detection

AI-based methods show glimpses of a paradigm shift in the assessment of IBD, by enhancing multiple facets of endoscopic lesion recognition, risk stratification, and standardization. Since the late 2010s, there have been a multitude of publications exploring the relevance and performance of AI-based tools either as an aid or as an independent diagnostic tool in IBD endoscopy, mostly with promising results. Thus, various computer-aided detection (CAD) modules, varying in training, design, and scope, have been developed and broadly tested [15].

Disease activity assessment is one of the primary objectives of a colonoscopy in IBD and is a key factor in selecting the therapeutic approach. However, the different severity scores have relatively high inter- and even intra-observer variabilities, with agreement rates ranging from a dismal 0.58 to a relatively good 0.80, according to a recently published meta-analysis [5]. In this light, finer standardization, especially in the mild-to-moderate disease severity spectrum and remission assessment, is desirable to avoid under- or overtreatment. Deep convolutional neural networks (CNNs) have demonstrated exceptional performance in detecting mucosal abnormalities, including ulcers, erosions, pseudopolyps, and strictures. For instance, the CAD system developed by a Japanese study group trained on over 26,000 colonoscopy images achieved AUROCs of 0.86 and 0.98 for identifying Mayo 0 and 0–1, respectively, supporting its utility in recognizing mucosal healing and quiescent or mild disease, with the byproduct of reducing interobserver variability [16]. Another group developed a CNN trained on over 40,000 images and 6800 biopsies from more than 2000 UC patients. This system predicted endoscopic remission with 90.1% accuracy and histologic remission with 92.9% accuracy, demonstrating that CNNs can infer histologic states, even without biopsies, thus signaling a potential cost- and time-reduction strategy [17].

The methods in which the AI models are designed also vary significantly, ranging from tech-savvy black box designs requiring large datasets to ingeniously crafted solutions based on empirical strategies, which could be trained on low volumes of patients. An example of the latter approach was designed by the Belgian group coordinated by Raf Bisschops, who introduced the Red Density algorithm, using a training set of 29 patients with IBD. This algorithm combined red–green–blue pixel color data and a training set for mucosal pattern recognition. Thus, beginning with the white light endoscopy image, the system generated a redness map based on red pixel propensity and was trained to extract vascular redness and detect the vascular pattern. As a result, Red Density correlated strongly with both Mayo (r = 0.76) and UCEIS scores (r = 0.74), allowing for real-time, objective disease activity measurement [18]. In contrast, a more conventional recurrent CNN model was designed by the group of Gottlieb et al. using 795 full-length colonoscopy videos and approximately 20 million frames to assign Mayo and UCEIS scores with high agreement with expert central readers (0.844 for Mayo and 0.855 for UCEIS) [19].

Detection of subtle or flat lesions, especially harboring islets of dysplasia of various degrees, remains a challenge in IBD surveillance. It is highly dependent on the observer’s experience and prone to underdiagnosis and inaccurate biopsy targeting. Moreover, adequate detection and characterization of dysplastic lesions can significantly alter therapy and the patient’s quality of life, especially in UC, and can lead to an indication for colectomy or proctocolectomy [20]. Maeda et al. demonstrated that repurposing AI systems initially designed for other outcomes, like EndoBRAIN-EYE, could identify low-grade dysplasia in longstanding UC patients during high-definition endoscopy, offering a major advance in cancer prevention [21]. A Japanese study group designed a CNN trained on over 800 non-magnified endoscopic images of 99 IBD lesions to distinguish adenocarcinoma or high-grade dysplasia from low-grade dysplasia, sporadic adenoma, or normal mucosa. The results were subsequently compared to non-expert and expert-level endoscopists. The AI system had a better sensitivity (72.5% vs. 70.5%) and accuracy (79% vs. 77.8%) when compared to the expert endoscopists, with the contrast increasing in comparison to non-experts [22]. Another study using a CAD-based solution (CADe Discovery™) included 52 patients undergoing virtual chromoendoscopy with virtual enhancement and compared the diagnostic performance between one blinded endoscopist and a second endoscopist assessing the CAD-detected lesions. The study reported similar sensitivity and specificity between the two, both exceeding 80%, albeit the figures were slightly lower for the AI-based system [23]. While to this point the results show good diagnostic prowess for AI solutions, real-life day-to-day practice data is lacking, and a sensible inference suggests that the addition of automatic detection tools could provide an even higher increase in diagnostic performance for non-expert operators.

A multicenter controlled study in Denmark also reported a significant increase in adenoma detection rate (ADR) when CADe was used, with a detection rate of 59.1% compared to 46.6% in the control group [24]. Encouraging results have been confirmed in a systematic review and meta-analysis by Makar et al. that included 28 RCTs involving 23,861 participants that demonstrated a 20% increase in ADR and a 55% decrease in adenoma miss rate with AI-assisted colonoscopy. CADe use also significantly increased adenomas per colonoscopy, primarily because of increased diminutive lesion detection also facilitated by an average 0.15 min prolongation of withdrawal time [25]. These findings are especially relevant in IBD surveillance due to the elevated risk of colorectal cancer in this population.

Additional studies confirm the robustness of CNNs. Stidham et al. demonstrated CNN-based grading of UC severity comparable to human experts (AUROC 0.97; κ = 0.84) [26]. Maeda et al. also validated an endocytoscopy-based AI system with 91% accuracy in identifying histologic inflammation [21].

### 3.2. Assessing Quality in Endoscopy

AI has also shown potential in the automated assessment of procedural quality metrics. Lui et al. assessed a novel AI-derived quality metric for colonoscopy called Effective Withdrawal Time (EWT), designed to better reflect the quality of mucosal inspection than standard withdrawal time (SWT). In a cohort of 350 colonoscopy videos, higher EWT was strongly associated with improved ADR, with each additional minute increasing the detection rate by 49%. EWT also outperformed SWT in predicting ADR, with a significantly higher area under the ROC curve (0.80 vs. 0.70, *p* < 0.01), suggesting its potential as a more accurate quality indicator [27].

In a complementary effort, Liu et al. developed and validated an AI-based system to objectively measure fold examination quality (FEQ) during colonoscopic withdrawal. The system’s FEQ scores correlated strongly with expert assessments (r = 0.871), historical ADR (r = 0.852), and withdrawal times (r = 0.727), confirming its accuracy. In a prospective observational study, the system significantly improved FEQ in colonoscopists with low baseline ADRs, as evaluated by both the AI model and human experts (*p* < 0.001). These findings highlight the system’s potential to support endoscopists in enhancing mucosal visualization and overall procedural quality [28].

Real-time AI integration during colonoscopy also improves procedural quality and lesion detection. The ENDOANGEL system, developed in China, uses CNNs to monitor withdrawal speed and mucosal inspection quality. In a randomized trial, it increased ADR from 8% to 16% and improved compliance with key procedural metrics like cecal intubation and withdrawal time [29].

The same system was also used for objectively assessing bowel preparation quality during colonoscopy. Using a deep CNN, the system demonstrated superior performance in a human–machine contest, achieving 93.33% accuracy with 120 images, outperforming all endoscopists. In 20 colonoscopy videos, ENDOANGEL achieved 89.04% accuracy and provided continuous, real-time bowel preparation scores every 30 s during the withdrawal phase. These results highlighted ENDOANGEL’s potential as a reliable, objective tool for consistent bowel preparation evaluation in clinical practice [30].

The most significant advances in AI-assisted endoscopy-based tools in IBD are summarized in Table 1.

## 4. Artificial Intelligence in Video Capsule Endoscopy

AI has emerged as a transformative force in video capsule endoscopy (VCE), especially in the context of small bowel CD, where automated lesion detection and interpretation streamlining are critically needed. VCE, while minimally invasive and ideal for visualizing mucosal inflammation, is often constrained by long reading times, high interobserver variability, and the risk of oversight due to the sheer volume of images. Deep learning, particularly through CNNs, has demonstrated its capacity to overcome these limitations by enabling high-accuracy detection of clinically relevant findings such as erosions, ulcerations, and strictures.

A foundational study by Aoki et al. introduced a CNN based on a Single Shot MultiBox Detector, trained on over 5000 annotated images of erosions and ulcerations. The model achieved an impressive AUC of 0.958, with 88.2% sensitivity and 90.9% specificity, validating its utility for routine VCE analysis [31]. The importance of this study lies not only in its accuracy metrics but also in its scalability potential; it evaluated over 10,000 images in under four minutes, hinting at AI’s feasibility for real-time applications. Importantly, the study noted that conventional methods relying on color-based detection often underperformed in identifying mucosal breaks, especially those mimicking surrounding tissue tones, thus underscoring AI’s advantage in nuanced pattern recognition over heuristic-based approaches. Ribeiro et al. developed a CNN trained on images from colon VCE, which achieved an accuracy of 99.6% and an AUROC of 1.00—indicating near-perfect classification. Furthermore, the model processed images at a rate of 90 frames per second, underscoring its feasibility for real-time clinical implementation [10].

Expanding on this foundation, another group presented a series of studies focusing specifically on CD-related lesions. Their deep learning model for ulcer detection was trained on 17,640 images and achieved patient-level AUCs ranging from 0.94 to 0.99, with accuracies exceeding 95%. The robustness of the model was further tested through leave-one-patient-out cross-validation, simulating real-world performance on unseen patient data, which significantly strengthened the validity of their findings [34]. Another study by the same group addressed a clinically underexplored but highly relevant feature in CD: intestinal strictures. The model differentiated strictures from both ulcers and normal mucosa across severity scales, with an AUC of 0.989 when distinguishing strictures from normal mucosa and 0.942 when distinguishing from ulcers [32]. This capability is particularly significant as strictures, often passable, can confound interpretation and pose risks of capsule retention. By integrating AI tools that can not only detect but also grade such findings, clinicians can potentially obtain an objective and quantitative assessment of disease burden, which is essential for treatment planning and monitoring. Furthermore, these models have been designed and tested using the PillCam™ platform—already prevalent in clinical settings—suggesting ease of adoption and relevance to current practice [33].

In addition to detection accuracy, the use of AI in VCE shows great promise in reducing interpretation time and interobserver variability, two longstanding challenges in gastrointestinal diagnostics. Aoki et al. further evaluated the clinical utility of their CNN-based system as a first screening tool, comparing readings by experts and trainees with and without AI assistance. While AI-supported readings reduced interpretation time by over 70% (from 12.2 to 3.1 min for experts and 20.7 to 5.2 min for trainees), the lesion detection rate remained statistically unchanged, emphasizing AI’s role as a viable first-pass filter [35]. Similarly, Brodersen et al. employed the AXARO^®^ AI framework, which condensed review images to just 2.1% of the original dataset while maintaining a diagnostic sensitivity of 96% for CD [36]. Their multi-center, blinded design strengthens the generalizability of results and reflects the feasibility of integrating AI systems into routine workflows without compromising diagnostic integrity. In another pragmatic approach, one study group evaluated an AI algorithm that removed poorly visualized frames from full VCE datasets, thereby reducing mean reading times by 35.6% with preserved diagnostic concordance [37]. The study emphasized that despite frame reduction, no lesions were missed, supporting the idea that quality over quantity in image review may enhance diagnostic precision. These workflow improvements are crucial given that current VCE evaluations often require over an hour per patient, leading to clinician fatigue and potential oversight.

Ultimately, the adoption of AI tools in VCE aligns with broader trends in precision medicine, enabling more standardized, efficient, and reproducible diagnostics. Tools like CNNs can mitigate known drawbacks of human scoring indices prone to subjective interpretation [38]. AI offers objective lesion quantification, segmentation by anatomical location, and consistency across time points—indispensable qualities for longitudinal disease tracking and therapeutic response monitoring. Moreover, multicenter pilot studies like that by Ferreira et al. highlight the feasibility of AI integration into novel capsule platforms like the PillCam Crohn’s Capsule, further suggesting a future where endoscopy interpretation becomes less about manual review and more about AI-augmented decision-making [33]. As these tools continue to mature and gain regulatory traction, their role will likely extend beyond mere detection into comprehensive disease activity indexing, paving the way for AI-assisted precision gastroenterology.

## 5. Artificial Intelligence in Ultrasonography

US is a valuable imaging tool for assessing IBD due to its non-invasiveness, real-time capabilities, and bedside availability. However, its operator dependency remains a challenge, introducing variability in diagnostic accuracy. AI has been proposed to improve the reliability and reproducibility of US evaluations, although no established consensus exists regarding its role in transabdominal intestinal US. As AI technologies evolve, their integration into clinical workflows may significantly enhance diagnostic consistency across different settings. Tagliamonte et al. highlighted the complementary roles of CNNs for image recognition and RNNs for temporal data analysis, both relevant for tracking disease progression over time [39].

A key parameter in evaluating IBD activity is bowel wall thickness (BWT), measured from the lumen/mucosa interface to the muscularis/serosa interface. Kumaralingam et al. developed an AI-based algorithm for automated BWT measurement, which showed a sensitivity of 90.29% and a specificity of 93.70% at the 2 mm BWT cutoff. An excellent agreement with expert assessments (ICC: 0.942) was also demonstrated. This approach enables reproducible whole-bowel evaluation and supports standardized monitoring, overcoming the limitations of manual measurement [40].

In a similar effort, Carter et al. developed a machine learning model using a pretrained CNN to detect BWT greater than 3 mm. This model achieved strong diagnostic performance, with an overall accuracy of 90.1%, sensitivity of 86.4%, and specificity of 94%, demonstrating its ability to identify abnormal BWT accurately. This model has the potential to standardize BWT assessments and reduce inter-operator variability, ultimately enhancing the quality of IBD monitoring [41].

Another relevant parameter is bowel wall vascularization, typically evaluated through color Doppler imaging or contrast-enhanced ultrasound (CEUS). Increased vascularity is associated with active inflammation, while normalization may indicate remission. Although AI has shown promise in analyzing structural features like BWT, its application in assessing vascularization remains underexplored, representing a promising area for future development [42].

### 5.1. AI for Fibrosis, Strictures, and Elastography in CD

One of the critical challenges in IBD imaging, particularly in CD, is the accurate differentiation between inflammatory and fibrotic strictures. Misclassification can lead to suboptimal treatment choices, such as administering anti-inflammatory therapy to a patient with predominantly fibrotic disease. In this context, AI-enhanced image interpretation offers a new layer of precision. Gu et al. explored the use of radiomics for analyzing IUS images. Retrospectively analyzing IUS images obtained during routine outpatient visits, the authors developed and tested radiomic-based and CNN-based models to distinguish between normal and abnormal images, with abnormal images defined as bowel wall thickness > 3 mm or bowel hyperemia with modified Limberg score ≥ 1, both being surrogate markers for inflammation. Their model, trained on 125 bowel segment images, achieved an AUC of 0.98 with 93.8% sensitivity and specificity—clearly outperforming a traditional CNN model, which achieved an AUC of 0.75 [43,44]. These findings underscore the superiority of radiomics-based texture analysis over conventional CNNs for detecting subtle IUS changes.

Another underappreciated element in CD imaging is perienteric fat, which often appears uniform on US but may carry pathophysiological significance. Sleiman et al. highlighted the possible role of perienteric fat in stricture formation, an area where AI could help detect and quantify changes that escape the human eye [45]. Beyond grayscale imaging, color Doppler imaging is being explored for AI-based assessment of vascularity patterns to assist in stricture characterization. A prospective study of 17 CD patients undergoing bowel resection assessed how IUS findings correlate with histopathological features. IUS showed high diagnostic performance for detecting stricturing (sensitivity 93%, specificity 86%) and penetrating complications (sensitivity 78%, specificity 92%). Notably, hyperechogenic spiculates were significantly associated with fibrosis, while increased vascular signals on color Doppler (Limberg score 3–4) correlated with active inflammation. IUS-measured wall thickness also strongly correlated with histologic thickness (r = 0.67). These results support IUS as a reliable, non-invasive modality for assessing structural and inflammatory changes in Crohn’s disease [46].

A 2021 international consensus study led by Novak et al. developed the International Bowel Ultrasound Segmental Activity Score (IBUS-SAS) to standardize the assessment of CD activity via IUS. Through a three-phase process involving 12 experts from 9 countries, 4 key sonographic parameters were identified: BWT, bowel wall stratification, color Doppler signal, and inflammatory mesenteric fat. The final IBUS-SAS demonstrated excellent inter-rater reliability, with an intraclass correlation coefficient (ICC) of 0.97, indicating its potential as a robust, non-invasive tool for consistent evaluation of segmental disease activity in Crohn’s disease [47].

A 2024 systematic review by Lu et al. examined 56 studies to assess the use of IUS in defining, diagnosing, and monitoring small bowel strictures in CD. While IUS demonstrated high diagnostic accuracy for detecting strictures, the review highlighted significant variability in definitions and technical parameters across studies. Importantly, IUS currently lacks sufficient precision to reliably differentiate between inflammatory and fibrotic strictures for clinical decision-making. The authors emphasize the need for standardized definitions, imaging protocols, and the development of a validated IUS-based index to enhance the assessment and management of stricturing Crohn’s disease [48].

Elastography has emerged as a promising non-invasive technique for assessing tissue stiffness, with applications in various clinical settings, including IBD. Cè et al. highlighted that while elastography offers advantages such as safety and cost-effectiveness, it faces challenges like operator dependence and low specificity. The incorporation of AI has the potential to mitigate these limitations by enhancing data acquisition and interpretation, thereby improving diagnostic performance and facilitating clinical integration [49]. Building upon these foundational insights, Demir et al. conducted a prospective study involving 60 IBD patients to evaluate the efficacy of 2D shear-wave elastography (SWE) in assessing disease activity. Their findings demonstrated a significant correlation between intestinal stiffness measurements and established clinical activity indices, such as the Crohn’s Disease Activity Index (CDAI) and the Mayo score [50]. These results underscore the potential of 2D-SWE as a valuable adjunct tool in the non-invasive evaluation of IBD activity. Together, these studies suggest that integrating AI with USE could enhance the accuracy and reliability of IBD assessments, paving the way for more personalized and effective patient management strategies [49,50].

### 5.2. AI-Enhanced Monitoring of Disease Activity

Assessing disease activity in IBD, particularly during transitions between active disease and remission, remains challenging. Contrast-enhanced ultrasonography (CEUS), offering real-time vascular imaging, is a valuable tool in this context. When combined with AI, it enables precise, dynamic monitoring. In a cohort of 127 CD patients, Medellin-Kowalewski et al. generated quantitative time–intensity curves that correlated well with standard disease severity markers. The CEUS parameters showed >85% sensitivity and specificity, suggesting strong diagnostic performance, especially when interpreted with AI support [51].

While transmural healing assessed by US is not yet a validated treatment target in clinical guidelines, it is increasingly being considered as a marker of deep remission, particularly in CD. Turner et al. have argued that its inclusion alongside endoscopic remission could refine treat-to-target strategies [52]. Kucharzik et al. followed 234 adult patients with active CD over 12 months and demonstrated that improvements in bowel wall thickness and vascularity, as seen on US, were correlated with significant reductions in C-reactive protein and Harvey–Bradshaw Index scores. These findings provide a compelling case for using US to monitor healing over time [53].

Alongside healing metrics, predicting treatment response is another major application for AI in IBD. Stafford et al. found that while earlier AI research focused on therapy response, more recent studies emphasize diagnostic precision. However, both directions remain crucial [54].

As proposed by Calabrese et al., a reduction in BWT (>25%, >2.0 mm, or >1.0 mm) and decreased color Doppler signal can serve as objective criteria for assessing therapeutic success. Incorporating these thresholds into machine learning models could automate response tracking, allowing clinicians to adjust therapy in near real time [55].

AI-supported US monitoring offers distinct advantages in the pediatric population —notably, its radiation-free, bedside availability—but current efforts are also directed toward expanding its use in adult care. As models grow more sophisticated, the integration of vascular, structural, and elastic parameters promises an increasingly granular and actionable portrait of disease dynamics.

The most significant advances in AI-assisted ultrasonography-based tools for assessing IBD activity and staging are summarized in Table 2.

## 6. Challenges and Limitations

AI holds transformative potential in enhancing endoscopic and ultrasound-based imaging for IBD. However, its implementation comes with several challenges and limitations that must be addressed for successful integration into clinical practice.

### 6.1. Data Quality and Standardization Issues

One of the primary challenges in developing robust AI models is the need for large, high-quality datasets. These datasets must encompass diverse patient populations, various stages of disease activity, and multiple imaging modalities, including endoscopy and ultrasound. Data acquisition in clinical settings, however, is often fragmented, with variability in imaging quality and protocols. For instance, ultrasound images may differ significantly depending on the operator’s expertise, the machine’s resolution, and the settings used during acquisition. Similarly, endoscopic imaging platforms from different manufacturers may produce varying results in terms of image clarity, color contrast, and lesion visibility. These inconsistencies can hinder the development of generalized AI models and limit their applicability across healthcare systems.

Moreover, the lack of standardized annotation protocols for imaging datasets further complicates the training of AI algorithms. Manual annotation is time-intensive and prone to interobserver variability, especially in subjective assessments such as lesion severity or disease activity scoring. Establishing uniform guidelines for data collection, annotation, and preprocessing is critical to ensure the reproducibility and reliability of AI models in IBD imaging [56].

### 6.2. AI Interpretability and Clinical Acceptance

The successful adoption of AI tools in clinical workflows requires a high level of trust and understanding from clinicians. However, the “black box” nature of many AI models often raises concerns regarding their interpretability [57]. Clinicians may be hesitant to rely on AI-generated results without a clear understanding of the underlying decision-making processes. This lack of transparency can impede the acceptance of AI tools, particularly in high-stakes scenarios such as differentiating active inflammation from fibrosis or predicting disease progression.

Integrating AI into real-world workflows also presents practical challenges. AI tools must seamlessly integrate with existing electronic health record systems and imaging platforms to avoid disrupting clinical efficiency. Additionally, there is a need for comprehensive training programs to familiarize clinicians with AI technologies and their potential applications in IBD management. Collaborative efforts between AI developers and healthcare providers are essential to design user-friendly interfaces and establish clear protocols for AI-assisted decision-making.

### 6.3. Ethical and Regulatory Considerations

The implementation of AI in IBD imaging also raises significant ethical and regulatory questions [43,58]. Ensuring patient data privacy is paramount, particularly when large datasets are shared across institutions for algorithm development. Data anonymization and secure storage protocols must be rigorously enforced to prevent breaches of sensitive information.

Another ethical concern is the accountability of AI-driven decisions. In scenarios where AI algorithms provide diagnostic recommendations or predict treatment outcomes, it is essential to delineate the responsibility between the clinician and the AI tool. Establishing clear guidelines for AI-assisted decision-making is crucial to maintain patient safety and trust [59,60].

From a regulatory perspective, the approval and deployment of AI-based imaging tools face several hurdles. Regulatory agencies must develop specific pathways to evaluate the safety, efficacy, and reliability of these technologies. This includes validating AI algorithms on diverse, real-world datasets and assessing their performance in prospective clinical trials. Harmonizing regulatory standards across different regions will also be critical to facilitate the widespread adoption of AI tools in IBD care.

Addressing these challenges will require concerted efforts from clinicians, researchers, industry stakeholders, and regulatory bodies. By overcoming these limitations, AI has the potential to revolutionize the imaging landscape for IBD, enhancing diagnostic precision, optimizing treatment strategies, and improving patient outcomes.

## 7. Future Directions

As AI continues to permeate clinical medicine, its role in IBD imaging—particularly in endoscopy and ultrasound—is rapidly evolving. Several key areas are poised for future development and integration into clinical practice, research, and trial design.

One major frontier is the fusion of AI with multi-modal data streams, including clinical, endoscopic, radiologic, histologic, genomic, and microbiome inputs. The concept of “endo-omics,” introduced by Iacucci et al., exemplifies this approach, wherein AI combines molecular data with imaging to predict therapeutic response in real-time during endoscopy [61]. Such integrations may enable hyper-personalized care strategies, tailoring treatment plans based on predictive models that synthesize heterogeneous patient data.

Another pivotal development is the automation of advanced endoscopic imaging interpretation. High-resolution modalities such as virtual chromoendoscopy and endocytoscopy are underutilized due to their interpretative complexity. However, AI-driven tools like the ELECT score and EndoBRAIN-UC have demonstrated high accuracy in correlating endoscopic and histological activity, even predicting long-term remission [21,62]. Future models may obviate the need for biopsies by reliably inferring histological inflammation from image data alone. In the realm of ultrasound imaging, AI shows promise in enhancing standardization and real-time disease monitoring. Predictive algorithms for bowel wall thickness, vascularity, and shear-wave elastography are being refined to facilitate point-of-care decision-making. AI-enhanced intestinal ultrasound could ultimately serve as a cost-effective, radiation-free alternative to frequent MRI or CT monitoring, particularly if tethered to telemedicine platforms.

VCE also stands to benefit substantially. Studies have shown that AI can dramatically reduce review time while improving the accuracy of detecting ulcers, strictures, and erosions. Future applications may include prognostic tools that predict therapeutic escalation or hospitalizations based on VCE-derived image patterns, thus transforming CE from a diagnostic to a predictive modality.

In clinical trials, AI is expected to reduce costs and enhance efficiency by automating eligibility screening through electronic health records (EHRs), standardizing outcome assessments via computer vision, and even generating synthetic control arms to reduce placebo allocation. These innovations may accelerate trial recruitment and reduce reliance on central reading, making trials more accessible and equitable.

Nonetheless, several challenges remain. Model interpretability, data privacy, and generalizability across institutions must be addressed. Standardization frameworks such as SPIRIT-AI and CONSORT-AI are essential to ensure ethical and scientifically sound implementation [63,64]. Additionally, prospective validation in diverse populations is required to move AI tools from proof-of-concept to routine clinical use.

## 8. Conclusions

Artificial intelligence is rapidly reshaping the landscape of imaging in inflammatory bowel disease. From endoscopic interpretation to cross-sectional imaging and intestinal ultrasound, AI has demonstrated its ability to enhance diagnostic accuracy, reduce interobserver variability, and support personalized therapeutic decisions. In the field of intestinal ultrasound, AI shows promise by transforming a highly operator-dependent technique into a reproducible and objective tool—with applications ranging from automated bowel wall thickness measurement to fibrosis assessment, vascularity quantification, and transmural healing monitoring.

Although most current applications remain in the exploratory or early clinical phases, results from machine learning models suggest a paradigm shift in IBD management. Future efforts should focus on integrating multimodal data (imaging, biomarkers, clinical scores) into unified AI frameworks, and on validating these tools in prospective, multicenter cohorts.

As AI systems evolve and become more accessible, their thoughtful integration into clinical workflows could redefine standards in IBD diagnosis, monitoring, and treatment optimization—ultimately improving patient outcomes through data-driven, individualized care.

## Figures and Tables

**Table 1:** Artificial intelligence advances in endoscopy.

Study	AI Application	Disease Context	Key Outcome
Japanese CAD System [17]	Mayo Score prediction from colonoscopy images	Ulcerative Colitis	AUROC: 0.86–0.98, reduced variability
Gottlieb et al. [19]	Video-based CNN for Mayo and UCEIS scoring	Ulcerative Colitis	Agreement with expert readers: 0.844–0.855
Maeda et al. [21]	EndoBRAIN-EYE for dysplasia detection	Ulcerative Colitis	Better sensitivity than experts (72.5% vs. 70.5%)
Aoki et al. [31]	CNN for lesion detection in VCE	Crohn’s Disease	AUC: 0.958, Sensitivity: 88.2%
Klang et al. [32]	CNN for ulcer/stricture detection	Crohn’s Disease	AUC: 0.94–0.99, accuracy > 95%
Ferreira et al. [33]	CNN with PillCam platform	Crohn’s Disease	High lesion detection accuracy and feasibility
Stidham et al. [26]	CNN grading for UC	Ulcerative Colitis	AUROC: 0.97; κ = 0.84
Lui et al. [27]	AI metric for Effective Withdrawal Time	General	Each min increased ADR by 49%
ENDOANGEL [29]	Quality control system	General	Increased ADR and real-time monitoring

AI—artificial intelligence; CAD—computer-assisted detection; CNN—convolutional neural networks; UC—ulcerative colitis; UCEIS—ulcerative colitis endoscopic index of severity; VCE—video-capsule endoscopy; ADR—adenoma detection rate.

**Table 2:** Artificial intelligence advances in ultrasonography.

Study	AI Application	Disease Context	Key Outcome
Kumaralingam et al. [40]	Automated BWT measurement	IBD	Sensitivity: 90.29%, Specificity: 93.7%, ICC: 0.942
Carter et al. [41]	CNN for BWT >3 mm detection	IBD	Accuracy: 90.1%, Sensitivity: 86.4%, Specificity: 94%
Gu et al. [44]	Radiomics-based model for abnormal IUS detection	Crohn’s Disease	AUC: 0.98. Sensitivity and specificity: 93.8%
Medellin-Kowalewski et al. [51]	CEUS for disease activity	Crohn’s Disease	>85% sensitivity and specificity
Novak et al. [47]	IBUS-SAS scoring system	Crohn’s Disease	ICC: 0.97, standardizes disease activity scoring
Demir et al. [50]	2D shear-wave elastography	IBD	Correlation with Mayo and CDAI scores
Calabrese et al. [55]	AI model for response tracking via BWT	IBD	BWT reduction > 2 mm signals treatment success

AI—artificial intelligence; BWT—bowel wall thickness; IBD—inflammatory bowel disease; CNN—convolutional neural networks; IUS—intestinal ultrasonography; CEUS—contrast-enhanced ultrasonography; IBUS-SAS—International Bowel Ultrasound Segmental Activity Score; CDAI—Crohn’s disease activity index.

## Data Availability

No new data was created in this article.

## Abbreviations

The following abbreviations are used in this manuscript:

IBD	Inflammatory bowel disease
CD	Crohn’s Disease
UC	Ulcerative colitis
US	Ultrasound
MES	Mayo Endoscopic Score
SES-CD	Simple Endoscopic Score for Crohn’s Disease
AI	Artificial intelligence
CAD	Computer-aided detection
CNN	Convolutional neural networks
ADR	Adenoma detection rate
EWT	Effective withdrawal time
SWT	Standard withdrawal time
FEQ	Fold examination quality
VCE	Video capsule endoscopy
BWT	Bowel wall thickness
IBUS-SAS	International Bowel Ultrasound Segmental Activity Score
SWE	Shear-wave elastography
CDAI	Crohn’s disease activity index
CEUS	Contrast-enhanced ultrasonography
